# PS-04 - EVALUATING RAPID COMBO LATERAL FLOW DEVICES FOR ACUTE RESPIRATORY INFECTION OUTBREAKS IN ADULT CARE HOMES IN THE SOUTH EAST: A SERVICE EVALUATION

**DOI:** 10.1093/pubmed/fdag046.053

**Published:** 2026-07-09

**Authors:** Dr Samuel Healy, Dr Arun Joshi, Dr Simon Ferris, Dr Eleanor Turner-Moss, Dr Carla Hobart, Dr Anand Fernandes

**Affiliations:** UK Health Security Agency; UK Health Security Agency; UK Health Security Agency; UK Health Security Agency; UK Health Security Agency; UK Health Security Agency

## Abstract

**Background:**

Out-of-season acute respiratory infection (ARI) outbreaks in care homes have previously relied on PCR testing to identify influenza, while in-season management has often relied on symptom criteria alone. SARS-CoV-2 and Influenza A+B Antigen Combo Rapid Tests (LFDs) may allow faster identification of respiratory pathogens and more informed outbreak management decisions.

**Objectives:**

To evaluate the economic benefit, feasibility and acceptability of using LFDs for ARI outbreaks in adult care homes in the South East compared with existing PCR-based processes.

**Methods:**

A pragmatic in-service cost comparison evaluated the LFD pathway against the existing PCR system, including direct cost comparisons and narrative assessment of wider health benefits. Qualitative data from surveys and focus groups involving care homes, Health Protection Teams (HPTs) and other stakeholders were analysed thematically to explore feasibility and acceptability. The survey was distributed to all care homes in the region that used LFDs when reporting an outbreak, with voluntary focus group participation from survey respondents.

**Results:**

LFDs had higher setup costs than PCR (£33,945 vs £24,030) but were substantially cheaper per outbreak (£15.85 vs £313.71). Cost savings would be realised after approximately 33 outbreaks, with potential direct savings of up to £14,000 annually across the South East. Wider benefits not captured in the cost comparison included faster and more appropriate antiviral prescribing. Thematic analysis identified consistently positive stakeholder feedback. LFDs were viewed as a valuable outbreak management tool that improved staff confidence and reassurance. HPTs also reported reduced time spent clarifying symptoms and making antiviral decisions. Operational barriers were noted including challenges with resupply and with spreadsheet-based reporting processes.

**Conclusion:**

LFDs represent a feasible, acceptable and potentially cost-saving alternative to PCR for ARI outbreak management in care homes across the South East region. Operational challenges, alongside regional differences in outbreak management and costs, should inform national implementation.

